# Marked Genetic Differentiation between Western Iberian and Italic Populations of the Olive Fly: Southern France as an Intermediate Area

**DOI:** 10.1371/journal.pone.0126702

**Published:** 2015-05-07

**Authors:** Barbara van Asch, Isabel Pereira-Castro, Fernando Trindade Rei, Luís Teixeira da Costa

**Affiliations:** 1 Instituto de Patologia e Imunologia Molecular da Universidade do Porto (IPATIMUP), Porto, Portugal; 2 Instituto de Ciências Agrárias e Ambientais Mediterrânicas (ICAAM), Universidade de Évora, Évora, Portugal; Federal University of Viçosa, BRAZIL

## Abstract

The olive fly, *Bactrocera oleae*, is the most important pest affecting the olive industry, to which it is estimated to cause average annual losses in excess of one billion dollars. As with other insects with a wide distribution, it is generally accepted that the understanding of *B*. *oleae* population structure and dynamics is fundamental for the design and implementation of effective monitoring and control strategies. However, and despite important advances in the past decade, a clear picture of *B*. *oleae*'s population structure is still lacking. In the Mediterranean basin, where more than 95% of olive production is concentrated, evidence from several studies suggests the existence of three distinct sub-populations, but the geographical limits of their distributions, and the level of interpenetration and gene flow among them remain ill-characterized. Here we use mitochondrial haplotype analysis to show that one of the Mediterranean mitochondrial lineages displays geographically correlated substructure and demonstrate that Italic populations, though markedly distinct from their Iberian and Levantine counterparts are more diverse than previously described. Finally, we show that this distinction does not result from extant hypothetical geographic limits imposed by the Alps or the Pyrenees nor, more generally, does it result from any sharp boundary, as intermixing is observed in a broad area, albeit at variable levels. Instead, Bayesian phylogeographic analysis suggests the interplay between isolation-mediated differentiation during glacial periods and bi-directional dispersal and population intermixing in the interglacials has played a major role in shaping current olive fly population structure.

## Introduction

The olive fly, *Bactrocera oleae* (Rossi, 1790), is a tephritid fly with a broad range of distribution and a significant economic impact. The species is thought to have originated in Africa, where it is still pervasive in Southern and Eastern areas, and spread to the Mediterranean basin, as well as South Central Asia. More recently, and presumably as a result of human intervention, it has also invaded North America [1]. *B*. *oleae* is the most important olive tree pest [2], causing production losses that are estimated to average approximately 15% yearly, but vary significantly depending on the region and year, and can be as high as 35% [3,4,5,6]. To the agriculture of the Mediterranean basin, where more than 95% of world olive production is concentrated, damaged caused by *B*. *oleae* translates into average annual losses in excess of 1 billion dollars.

In the Mediterranean, olive fly control has relied mainly on chemical insecticides. However, growing concerns over environmental damage, human health and the development of insect resistance are inducing a gradual shift towards more integrated pest control approaches. Under these circumstances, it is now generally accepted that a comprehensive understanding of olive fly's population structure and dynamics is crucial for the design of effective management and control strategies [7,8,9]. Previous genetic studies have established that there are three distinct Old World populations of *B*. *oleae*, corresponding to the original regions of dispersal: Sub-Saharan Africa, South Central Asia and the Mediterranean basin [10]. There is also good evidence for the existence of population substructure in the latter region, but we are still far from a comprehensive view. Indeed, earlier studies [11,12] suggested three Mediterranean subpopulations ("Eastern", "Central" and "Western"), but pointed to substantial population admixture; later analyses, on the other hand, suggested deep separation of the mitochondrial DNA (mtDNA) lineages found in the three areas [13,14]; and two very recent studies [7,8], though providing further support for the distinction between populations, again raised the possibility of significant gene flow between them.

In a previous study [14], we addressed the issue of the separation between Iberian (Western Mediterranean) and Italic (Central Mediterranean) olive fly populations using mtDNA analysis, and presented evidence of a deep split between them. This result, in turn, raised the questions of where the "boundary" between the two populations could be and to what extent do they intermix. In order to answer those questions, we have now extended our mtDNA analysis to samples collected in a ~1,000 km long swath of territory ranging from Eastern Spain to the Southern end of Liguria (Italy).

## Materials and Methods

### Olive fly samples

Olive flies were collected at or near 31 different localities, of which 10 across Portugal, four in Spain, 10 in France, six in Italy's Northern region of Liguria and one in Palestine (Table 1) by a combination of methods. Collections took place in public places for which permissions were not necessary, with three exceptions: in Nossa Senhora de Machede, on a property belonging to the corresponding author (LTC); in Valverde, on University of Évora's property; in Berqin, on a farm associated with Canaan Fair Trade (Kufor Qud Road, Burqin, Jenin, West Bank, Palestine), who graciously provided the olives. In most locations, olives were picked (preferably in groves not subject to chemical treatment) and stored in plastic bags, with emerging larvae, pupae and adults being collected periodically; a few larvae were also obtained by dissection of infested olives. In some locations, McPhail traps baited with a 5% solution of ammonium dihydrogen sulfate (Sigma-Aldrich) were placed in olive groves for periods of 1–2 weeks and inspected every 1–2 days. Trapped adults were collected and washed once in 70% ethanol before storage. All individuals were stored at -20°C in 70% ethanol until DNA extraction.

**Table 1:** *B*. *oleae* sample collection sites.

Area	Nearest Locality	Coordinates
Iberia (West)	Cabeça Gorda	37° 55' N; 7° 49' W
	Elvas	38° 52' N; 7° 16' W
	Évora	38° 35' N; 7° 55' W
	Guarda	40° 32' N; 7° 15' W
	Nossa Senhora de Machede	38° 35' N; 7° 47' W
	Oriola	38° 19' N; 7° 49' W
	Redondo	38° 39' N; 7° 38' W
	Tremês	39° 22' N; 8° 45' W
	Valverde	38° 32' N; 8° 01' W
	Vidigueira	38° 14' N; 7° 48' W
Iberia (East)	Banyeres del Penedès	41° 17' N; 1° 35' E
	La Jonquera	42° 24' N; 2° 52' E
	Oropesa del Mar	40° 08' N; 0° 08' E
	Vilajoan	42° 10' N; 2° 55' E
France (West)	Bages	43° 07' N; 2° 57' E
	Le Boulou	42° 31' N; 2° 49' E
	Les Matelles	43° 44' N; 3° 49' E
	Narbonne	43° 10' N; 2° 59' E
	Pollestres	42° 38' N; 2° 53' E
	Saint-Jéan-de-Védas	43° 34' N; 3° 50' E
France	Arles	43° 40' N; 4° 38' E
France (East)	Carpre	43° 45' N; 7° 23' E
	Nice	43° 43' N; 7° 16' E
	Vidauban	43° 25' N; 6° 27' E
Italy (Liguria)	Castello d'Invrea	44° 22' N; 8° 37' E
	Ponzano Magra	44° 08' N; 9° 55' E
	Sussisa	44° 24' N; 9° 07' E
	Terzorio	43° 51' N; 7° 55' E
	Ventimiglia	43° 48' N; 7° 36' E
	Vezzi Portio	44° 13' N; 8° 22' E
Palestine	Berqin	32° 28' N; 35° 15' E

### Selection of polymorphic mitochondrial DNA regions

Sections of mtDNA selected for amplification and sequencing were based on those used in our previous study [14], with adaptations (Table 2). Briefly, analyses centered on two of the five regions used in that study (total length = 1,826 bp; 11.5% of *B*. *oleae*'s complete mitochondrial genome), as they allowed a good discrimination between the three Mediterranean mtDNA lineages previously identified (with at least seven mutation steps separating them). For haplotypes of the P lineage, two other regions were included (Table 2), to allow a finer resolution of sublineages.

**Table 2:** *B*. *oleae* mitochondrial DNA segments analysed in this study.

Segment	Use	Primers (5’- 3’)		Sequence read (maximum common region)	
		Forward	Reverse	mtDNA coordinates*	Genes (% bp)
Segment 01	All analyses	CTAAGGAAATCACCCTATTTC	TTTGATTAACTTAAAGCCTTGCGTCAATAATGATATTAGACTGC	522–1189	ND2 (65.3)
Segment 03	All analyses	ATTCCCCAGCAATATTATGAGAATATGGCAGATTAGTGCAATG	CAATACTTGCTTTCAGTCATCGAGATTTCGGTTTGTATATGAG	2512–3670	COX1 (29.3), tRNA-Leu and COX2 (92.5)
Segment 05	P sublineagediscrimination	CCATTTTAACAGTATACCAATG	TATAAATAGAAGTATAAATGGAG	4917–5550	COX3 (80.45) and tRNA-Gly (87.7)
Segment 08b	P sublineagediscrimination	TCCTCAGTAAGTTAAAGTTATCAACCGTTCATAAGTAATATTCG	GAATTGTATGATTTCAAGAGG	9268–9858	ND4 (17.1), ND4L, tRNA-Thr and tRNA-Pro (7.6)

*Numbering according to the *B*. *olea* reference mitochondrial genome (Genbank accession AY210702).

### DNA extraction, amplification and sequencing

Total DNA was extracted using a standard SDS/Proteinase K method. Amplifications of the various mtDNA segments (Table 2) were performed in 25 μL reactions containing 10 ng of genomic DNA, 75 mM Tris—HCl (pH 8.8), 20 mM (NH_4_)_2_SO_4_, 0.01% (v/v) Tween 20 (Fermentas), 1.5 mM MgCl_2_ (Fermentas), 0.25 mM of each deoxy-NTP (Fermentas), 175 ng of each primer (Metabion) and 2.5 U of Taq DNA polymerase (Fermentas). The cycling protocol for segment 5 was: 95°C for 5 min; 3 cycles of 95°C for 30 s, 61°C for 1 min and 72°C for 1.75 min; 3 cycles of 95°C for 30 s, 58°C for 1 min and 72°C for 1.75 min; 3 cycles of 95°C for 30 s, 55°C for 1 min and 72°C for 1.75 min; 38 cycles of 95°C for 30 s, 58°C for 1 min and 72°C for 1.75 min; and 72°C for 5min. For the other segments, it was: 95°C for 5 min; 40 cycles of 95°C for 30 s, 58°C for 1 min and 72°C for 1.5 min; and 72°C for 5 min. Following purification with ExoI (Fermentas) and SAP (Fermentas), PCR products were used in sequencing reactions ("Sanger sequencing"). Part of the sequencing was outsourced from Macrogen Inc., and part was performed with the Big Dye Terminator Cycle Sequencing Kit (Applied Biosystems) and the following cycling protocol: 96°C for 3 min; 35 cycles of 95°C for 15 s, 56°C for 15 s and 60°C for 2 min; and 60°C for 5 min. Sequencing reaction products were purified using Sephadex G-50 micro-spin columns (GE Healthcare) and sequenced in an ABI Prism 3130 XL sequence analyzer (Applied Biosystems). Primers used in amplification or sequencing are listed in Table 2.

### Sequence and statistical analyses

Electropherograms were inspected using Chromas 1.45 (Technelysium Pty Ltd). Sequences were aligned and cropped to the maximum overlap for each segment and concatenated using CLC Main Workbench 6 (CLC Bio). File conversions were performed using FaBox [15] and Format Converter v2.2.5 available at http://www.hiv.lanl.gov/content/sequence/FORMAT_CONVERSION/form.html. Calculations of pairwise F_ST_, haplotype and nucleotide diversity, as well as the Mantel test were performed using Arlequin software v3.5.1.3 [16]. Median-joining phylogenetic networks were constructed using the Network software v4.612 available at www.fluxus-engineering.com, using default parameters in all calculations [17]. Six cases of suspected heteroplasmy were identified, which did not interfere with lineage and sublineage assignement, but led us to exclude the corresponding samples from at least some of the computations. Publicly available sequences with accessions GU108459, GU108470, GU108464, GU108471, GU108465, GU108461, GU108460, GU108472, GU108473, GU108479, GU108475 and AY210702 were also used in the analysis, the latter as *B*. *oleae*'s mtDNA reference sequence.

A detrended canonical correspondence analysis (DCA) was initially performed to determine the type of response associated to the explanatory variables (sampling regions, longitude and latitude) in the olive fly haplotype distribution. A gradient length <3 was obtained, indicating a linear response to these variables. Redundancy analysis (RDA) was therefore performed to analyze the relationships between explanatory variables and response variables (olive fly clades). Samples belonging to the O clade were excluded from the analysis due to their small number, and other data were transformed using the formula Y = log(y+1), to eliminate zero values. The significance of the fraction of variability attributable to explanatory variables was determined by Monte-Carlo permutation analysis. DCA and RDA analysis were performed using Canoco for Windows version v.5 (Microcomputer Power, USA) [18].

Phylogenetic analyses were implemented in the BEAST1.8 software package [19,20] using the GTR+I model of sequence evolution (selected using the Akaike Information Criterion with correction) with mutation rates and p_Inv_ as estimated by jModeltest [21] with a lognormal relaxed clock [22]. For phylogeographic analyses, a discrete trait (location) symmetric substitution model with a strict clock was also used. Given the results of Nardi *et al*. [13], a coalescent tree prior with exponential population growth, with priors on the age of the most recent common ancestor of Mediterranean samples, population size and population growth rate based on the results of the same study were used. To ensure convergence, two independent runs of 40 million generations (with a burn-in of 1 million and sampling every 1,600) were performed for each analysis. Trees were summarized and annotated with TreeAnnotator v1.8 and visualized using FigTree 1.4 (http://tree.bio.ed.ac.uk/software/figtree/) and GIMP2.

## Results

### Olive fly mtDNA diversity in the Italic peninsula is higher than previously described

**Fig 1:**
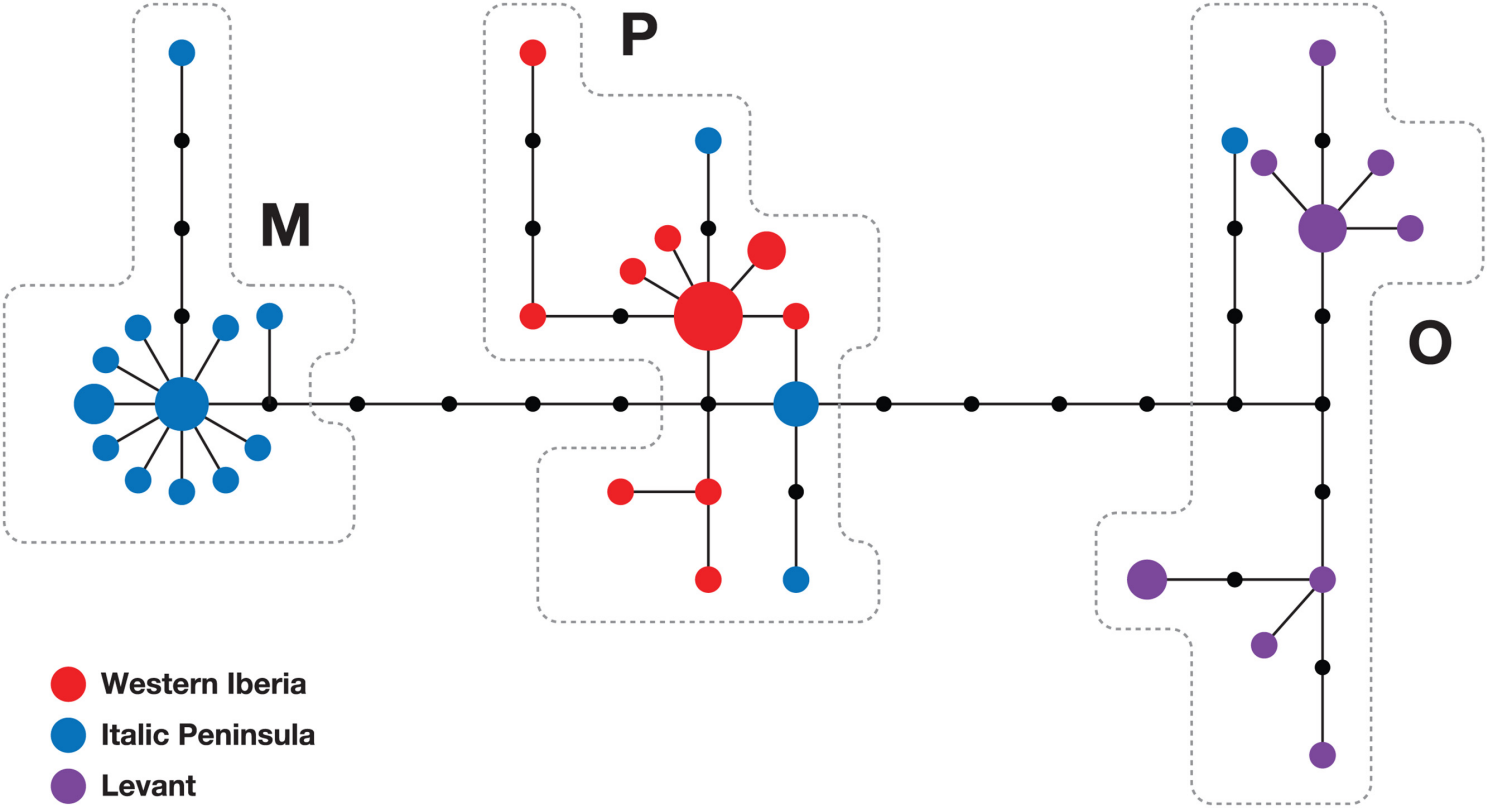
Olive fly mtDNA diversity in the Italic peninsula, Western Iberia and the Levant. Median-joining phylogenetic network of *B*. *oleae* mtDNA haplotypes found in 18 samples from Western Iberia (red), 27 from the Italic peninsula (blue) and 14 from the Levant (purple). Circles represent haplotypes, black circles represent unobserved intermediate haplotypes, the length of the connections is proportional to the number of mutational steps that separate the haplotypes, and the size of the circles is proportional to the frequency of the haplotype.

The main objective of this study was to investigate the geographic structure of the previously identified split between Italic and Iberian olive fly populations [14]. Considering the limited number of Italic sequences publicly available for analysis, we started by obtaining and analysing mtDNA sequences from the Italic peninsula. Though mitochondrial haplotype analysis is one of the most powerful and popular approaches to determine phylogenetic relationships between populations, its effectiveness naturally depends on the variability of the mtDNA fragments analysed, which, in the case of *B*. *oleae*, requires the use of relatively long stretches of mtDNA [23]. For this reason, we chose to analyse a 1.8 kb fraction of mtDNA harboring at least 7 differences between the Iberian and Italic samples previously studied [13,14]. Furthermore, based on the working hypothesis that the two large mountain ranges (the Pyrenees and the Alps) separating the Iberian and Italic peninsulas were the best candidates for a "natural border" between populations, we focused on Northwestern Italy. We therefore analysed 24 samples collected from six locations spread throughout Liguria, as well as 10 samples from Palestine, and compared their sequences to those from Western Iberian flies [13,14]. The results obtained confirm a clear distinction between Iberian and Italic *B*. *oleae* populations (F_ST_ = 0.47), as illustrated by the haplotype network (Fig 1). The high F_ST_ value found also demonstrates the validity of our choice of mtDNA sequences for studying olive fly population structure in Central and Western Mediterranean basin. Additionally, these results reveal olive fly mtDNA diversity in the Italic peninsula is higher than previously suspected, as all three Mediterranean mtDNA lineages were detected in the Ligurian samples (Fig 1). This is in sharp contrast to the results obtained in Western Iberia, where all 18 samples analysed belong to the same lineage, or around the Levantine region, where the 4 published sequences [13] and the 10 sequences obtained in this study also fall into a single (distinct) lineage. The three Mediterranean lineages were earlier referred to as Western, Central and Eastern Mediterranean or, alternatively, as Mauro-Iberian, Italo-Aegean and Levantine [14]. However, given our observations from Liguria, it seems more advised to use a less geographically-dependent nomenclature. In the remainder of this report they will therefore be referred to as the P, M and O lineages, designations that solely reflect the locations where they were first observed (Paradela, Monteccuco and Osmanyie, respectively).

### Phylogeographically-correlated substructure in the P mtDNA lineage

**Fig 2:**
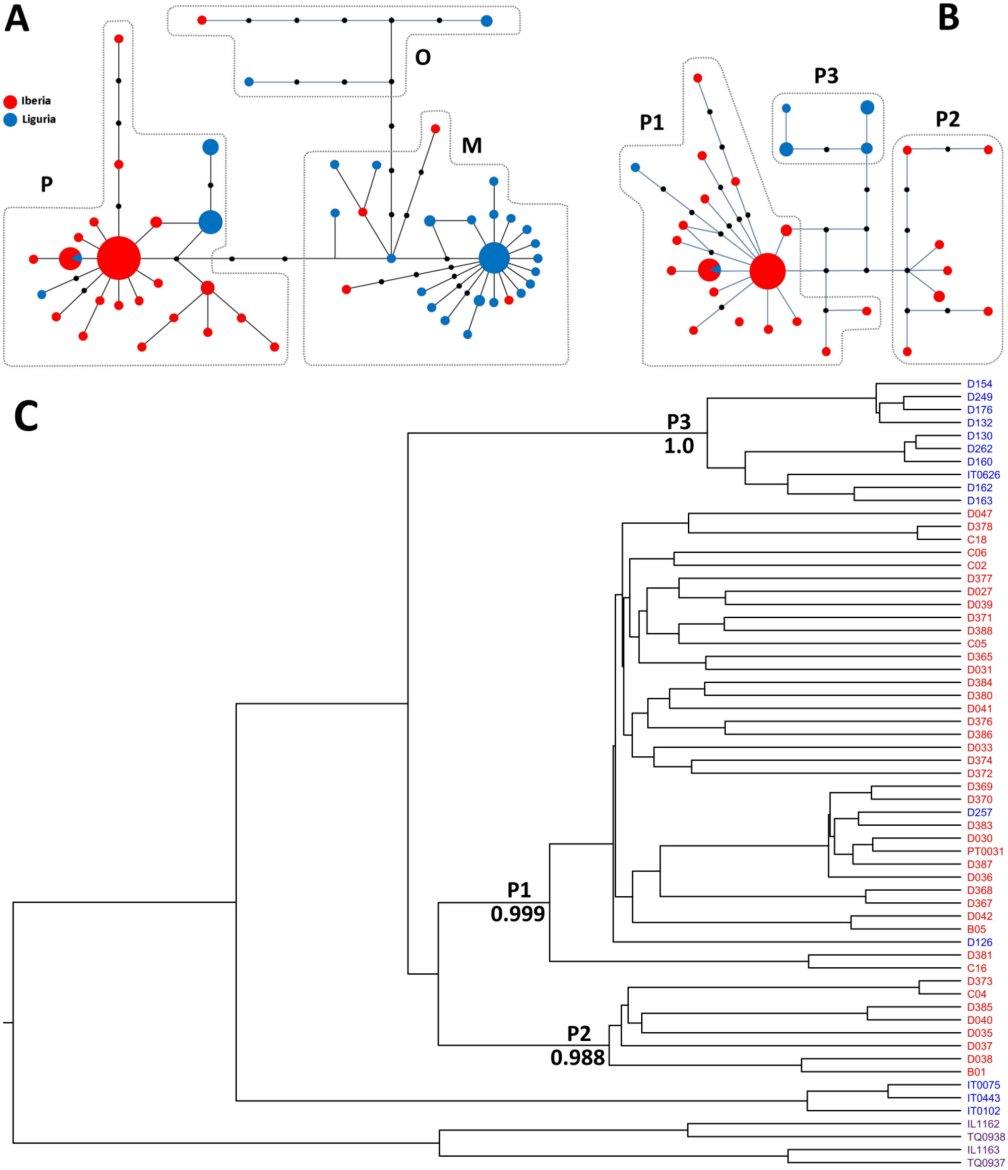
The P mtDNA lineage displays phylogeographically-correlated substructure. A) and B) Median-joining phylogenetic networks of *B*. *oleae* mtDNA haplotypes found in Iberia (red) and Liguria (blue) constructed using (A) two mtDNA segments (1,826 bp) or (B) four mtDNA segments (3,051 bp) of haplotypes belonging to the P lineage. Circles represent haplotypes, black circles represent unobserved intermediate haplotypes, the length of the connections is proportional to the number of mutational steps that separate the haplotypes, and the size of the circles is proportional to the frequency of the haplotype. C) Maximum clade credibility phylogenies for *B*. *oleae* mtDNA haplotypes found in Iberia (red), Italy (blue) and the Levant (purple) obtained using four mtDNA segments (3,051 bp).

The results described in the previous section prompted us to increase the numbers of Iberian and Ligurian samples analysed to obtain better estimates of the contributions of each lineage to the populations. As shown in Table 3 and Fig 2A, the large majority of the Iberian haplotypes analysed (43/48; 89.6%) belonged to the P lineage. Among Italic samples, on the other hand, though there was a clear predominance of M lineage haplotypes (34/ 48; 70.8%), the P lineage was also frequent (22.9%). This seemed to indicate that the split between Iberian and Italic populations is less pronounced than previously thought. Intriguingly, however, we observed that the Ligurian and Iberian samples seemed to have distinct distributions within the P lineage, with 81.8% (9/11) of the former carrying either haplotype IT0626 (previously found in Sicily) or a derivative thereof (D132). This led us to hypothesize they might belong to a third P sublineage, previously unrecognized. To further investigate this issue, we analysed two additional mtDNA segments (Table 2), harboring variants previously found in Sicily but not in the Western Mediterranean [13], to determine if they could help to dissect the substructure of the P lineage. The results obtained (Fig 2B and 2C) indeed confirmed that lineage P can be subdivided, with Bayesian analysis indicating very high support (>98.8%) for clades P1, P2 and P3, and showed that all nine Ligurian samples with haplotypes IT0626 or D132 fall into P3, whereas the 43 Iberian P haplotypes belong to either P1 or P2. This demonstrates the P lineage has geographically-correlated substructure and that the split between Iberian and Italic populations is very pronounced, as the predominant clades found in each of the two peninsulas have only a limited presence in the other (Table 3, Fig 2).

**Table 3:** Mitochondrial haplotype distributions in Liguria and Iberia showing the observed absolute frequencies of each lineage (or sublineage).

	mtDNA (sub)lineage				
	P1	P2	P3	M	O
Liguria	2	0	9	34	3
Iberia	35	8	0	4	1

### No sharp population boundary between Northwestern Italy and Western Iberia

**Fig 3:**
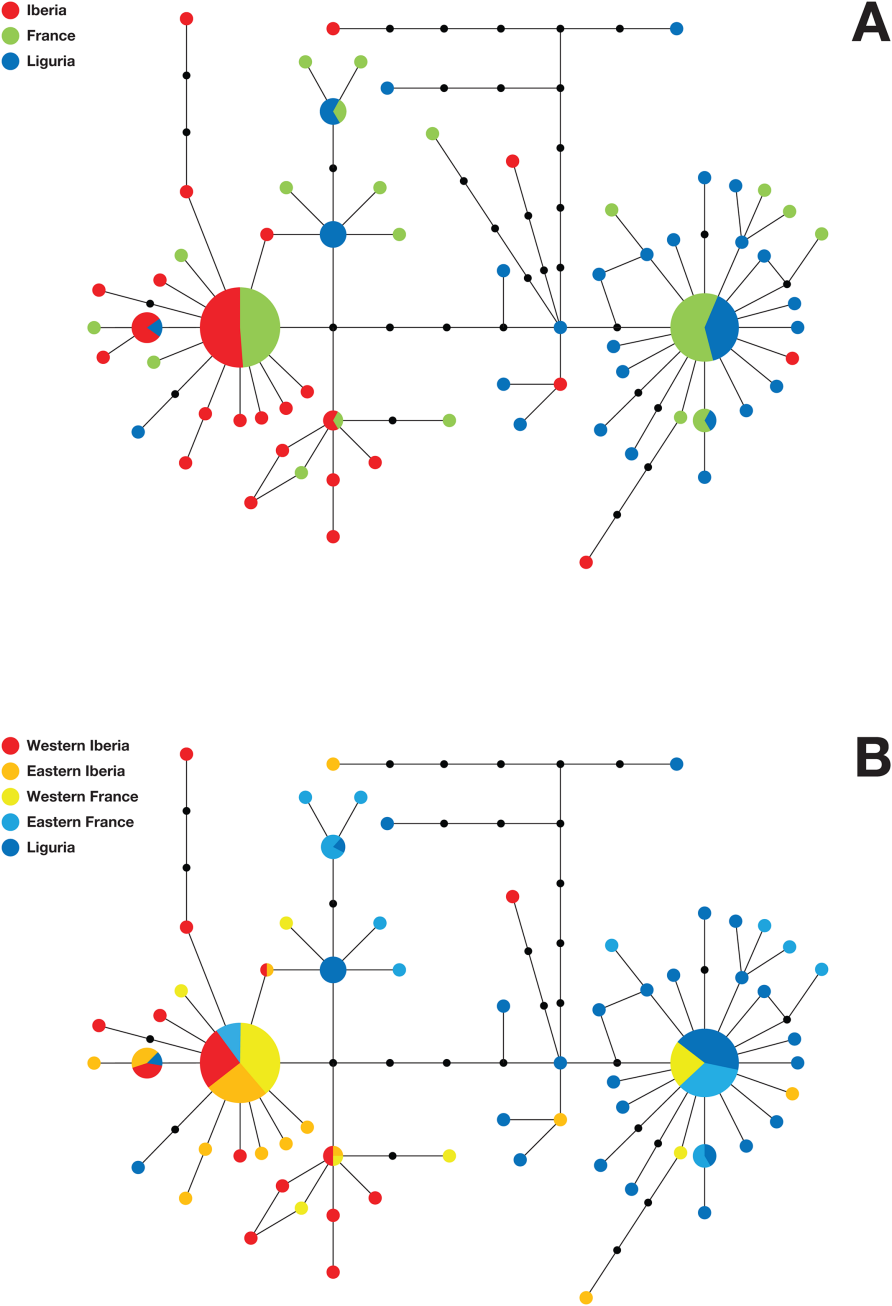
Geographic structure of olive fly population transition between Northwestern Italy and Western Iberia. Median-joining phylogenetic networks comparing *B*. *oleae* mtDNA haplotype distributions in Iberia, France and Liguria. A) Iberia (red), France (green), and Liguria (blue); B) Western Iberia (red), Eastern Iberia (orange), Western France (yellow), Eastern France (light Blue), and Liguria (blue). Circles represent haplotypes, black circles represent unobserved intermediate haplotypes, the length of the connections is proportional to the number of mutational steps that separate the haplotypes, and the size of the circles is proportional to the frequency of the haplotype.

**Fig 4:**
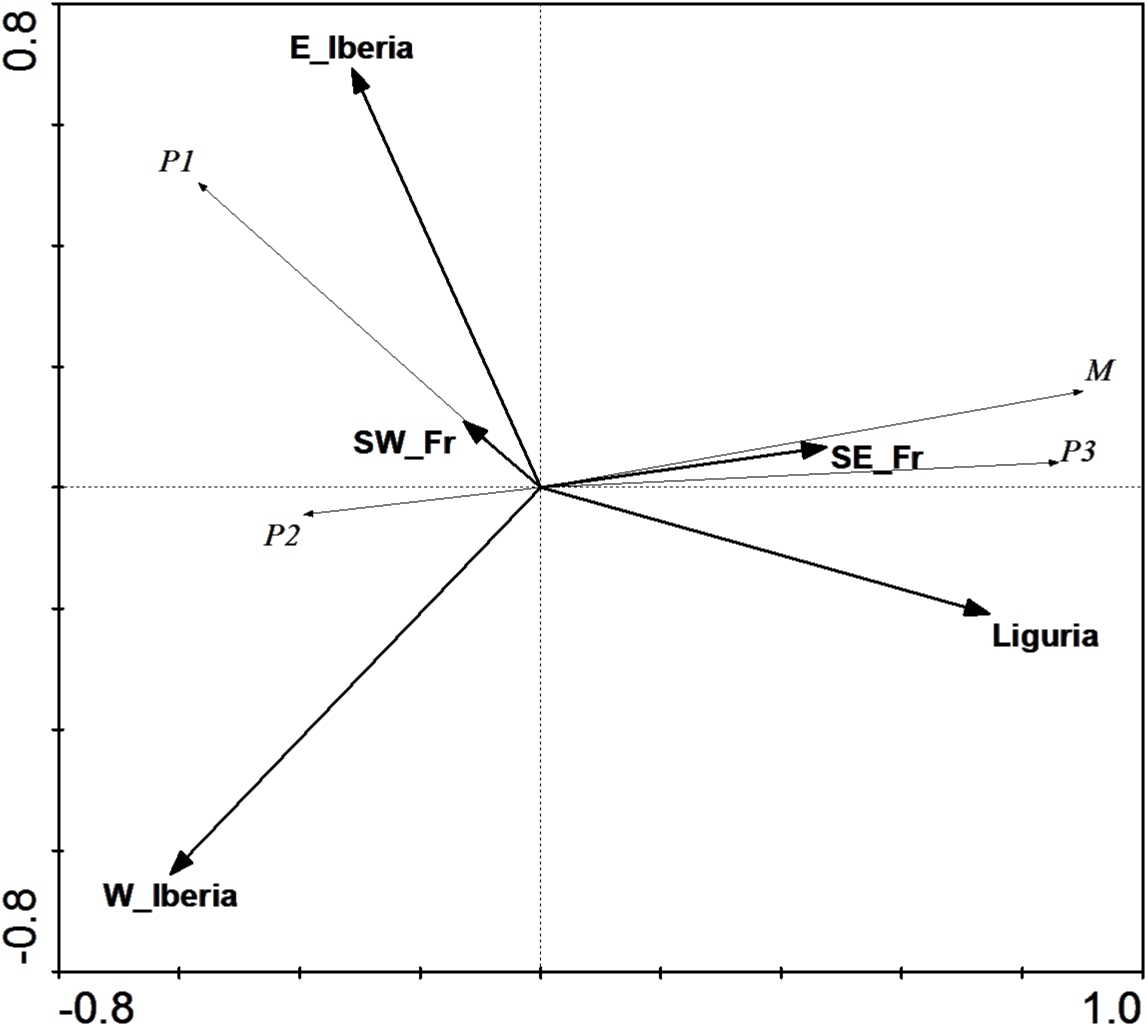
Redundancy analysis (RDA) with sampling regions as explanatory variables. RDA of olive fly mitochondrial haplotype distributions using lineage frequencies as response variables and sampling regions as explanatory variables. These explained 68.4% of the lineage distribution variability (Monte Carlo test, P<0.005). W_Iberia—Western Iberia; E_Iberia—Eastern Iberia; SW_Fr—Southwestern France; SE_Fr—Southeastern France.

**Fig 5:**
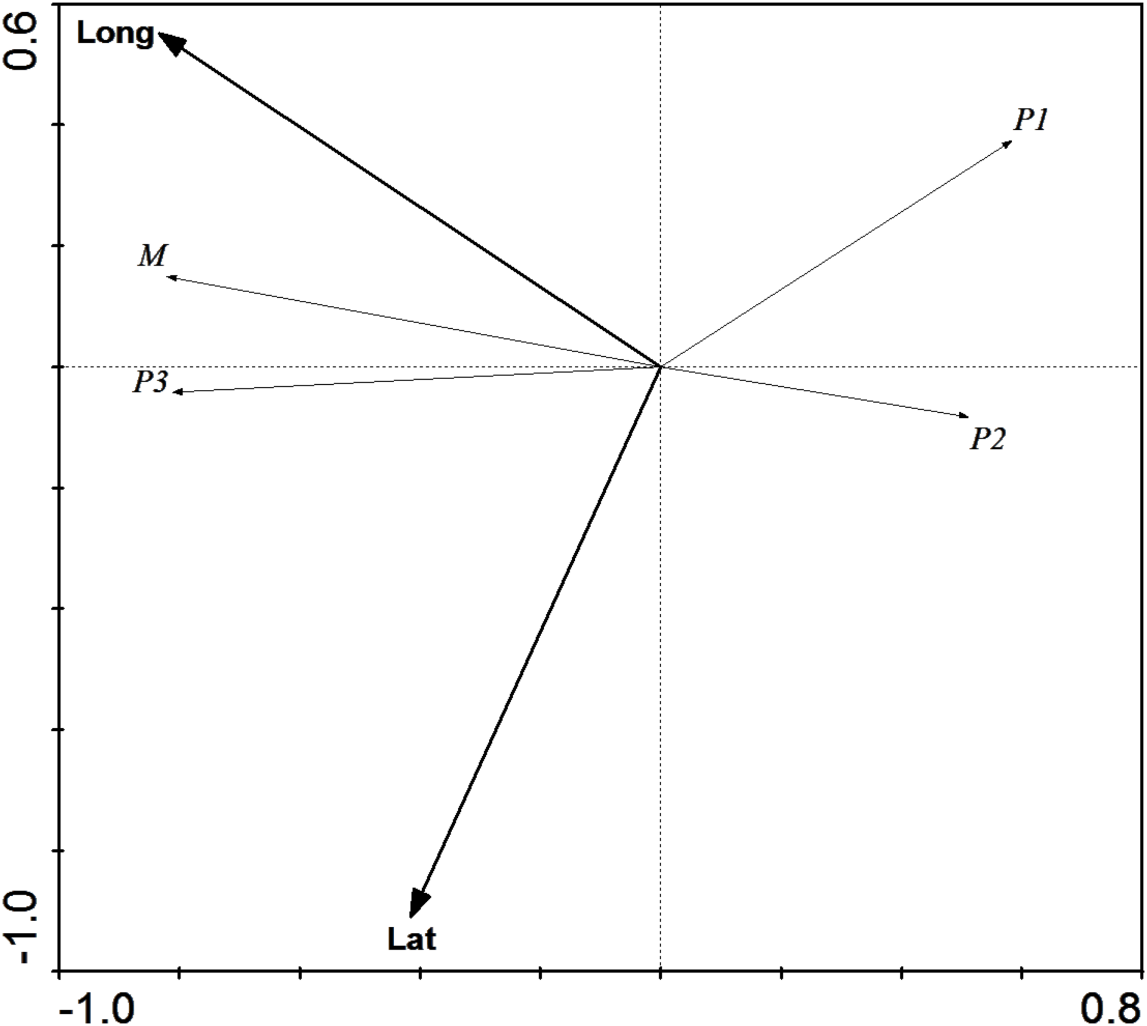
Redundancy analysis (RDA) with geographic coordinates as explanatory variables. RDA of olive fly mitochondrial haplotype distributions using latitude (Lat) and longitude (Long) as explanatory variables and lineage frequencies as response variables. Longitude alone explained 52.0% of lineage distribution variability (Monte Carlo test, P<0.005).

To determine the geographic structure of the split between Iberian and Italic populations we proceeded to compare them to a set of 56 samples collected throughout Southern France and the 14 available Levantine sequences ([13] and this study). A total of 77 haplotypes, defined by 88 sequence variants were identified (S1 Table). As shown in Fig 3A, Tables 4 and 5, the haplotype distribution within the French sample set was found to be intermediate between those of the Iberian and Italic peninsulas, suggesting that both the Alps and the Pyrenees act as barriers to olive fly's dispersal. However, upon closer inspection, no marked differences were observed between the haplotype distributions in Southeastern France and Liguria, on the one hand, and Southwestern France and Northeastern Iberia on the other (Fig 3B), as confirmed by redundancy analysis (Fig 4) and pairwise F_ST_s (Table 5), demonstrating that, in fact, neither of the two mountain ranges acts as a barrier. Instead, significant genetic differentiation was detected between Western France and both Eastern France and Western Iberia (Fig 4 and Table 5). Together with the observed lineage distributions (Table 4), this raised the possibility that a geographically-correlated gradual transition was responsible for the sharp differences in population structure between Iberia and Northwestern Italy. However, redundancy analysis (RDA) revealed that, despite a high correlation with longitude (Fig 5), geographical position cannot be considered the only major determinant of population structure.

**Table 4:** MtDNA sequence diversity in sample sets from different geographical locations. n—number of samples (het—number of samples with suspected heteroplasmy), h—number of haplotypes, H—haplotype diversity, and π—nucleotide diversity.

					% of samples in haplogroup				
Sample set	n (het)	h	H	π	P1	P2	P3	M	O
Iberia (total)	48	25	0.88	0.0020	72.9	16.7	0.0	8.3	2.1
France (total)	56 (2)	22	0.83	0.0027	35.7	5.4	12.5	46.4	0.0
Liguria	48 (1)	28	0.94	0.0029	4.2	0.0	18.8	70.8	6.3
France (SE)	24 (1)	12	0.87	0.0027	12.5	0.0	20.8	66.7	0.0
France (SW)	24 (1)	8	0.70	0.0021	54.2	12.5	4.2	29.2	0.0
Iberia (E)	24	15	0.89	0.0023	75.0	8.3	0.0	12.5	4.2
Iberia (W)	24	14	0.88	0.0017	70.8	25.0	0.0	4.2	0.0

**Table 5:** Pairwise F_ST_s between olive fly sample sets. Values indicating genetic differentiation are shown in bold.

	Iberia (total)	France (total)	Liguria	France (SE)	France (SW)	Iberia (E)	Iberia (W)
Iberia (total)	0						
France (total)	0.17075*	0					
Liguria	0.38247*	0.08131**	0				
France (SE)			(0.0)***	0			
France (SW)			0.22294*	0.19312**	0		
Iberia (E)			0.32310*	0.30215*	0.01463	0	
Iberia (W)			0.41076*	0.40550*	0.07751**	0.01038	0

*P < 0.0001;

**P < 0.02;

*** Negative estimated value.

### Bayesian analyses suggests ancient olive fly dispersal in both westwards and eastwards directions

**Fig 6:**
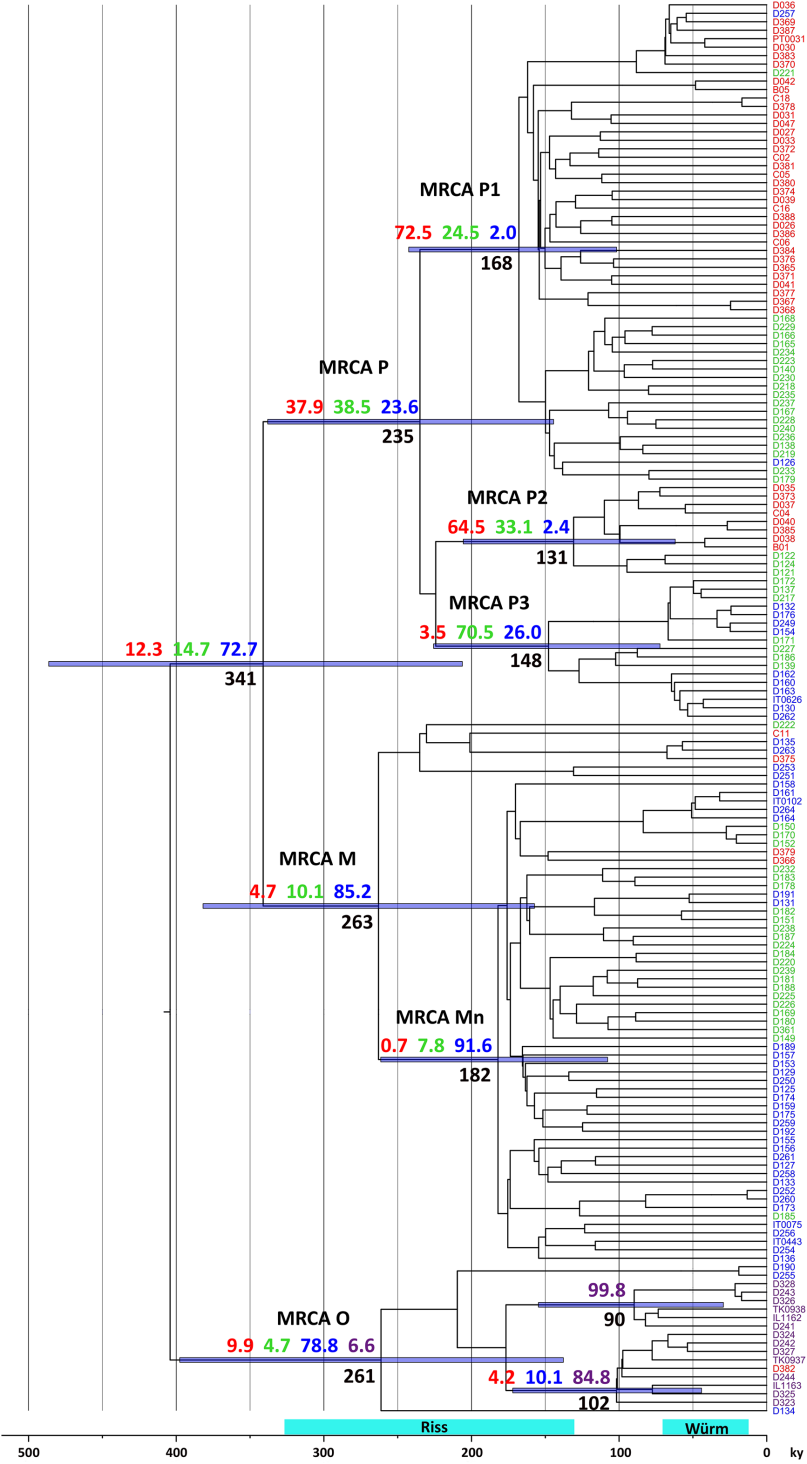
Bayesian phylogeographic analysis using the full sample set suggests both eastward and westward ancient olive fly dispersal in the Mediterranean basin. Maximum clade credibility phylogenies for olive fly partial mtDNA. Numbers indicate ages (in thousands of years BP) and probabilities of location (in percentage) for key, well-supported (posterior >0.95) nodes (including MRCAs of several clades). Bars represent confidence intervals (95% HPD) for ages. Red—Iberia, Green—France, Blue—Italy, Purple—Levant. The last two glacial periods are indicated by light blue bars.

**Fig 7:**
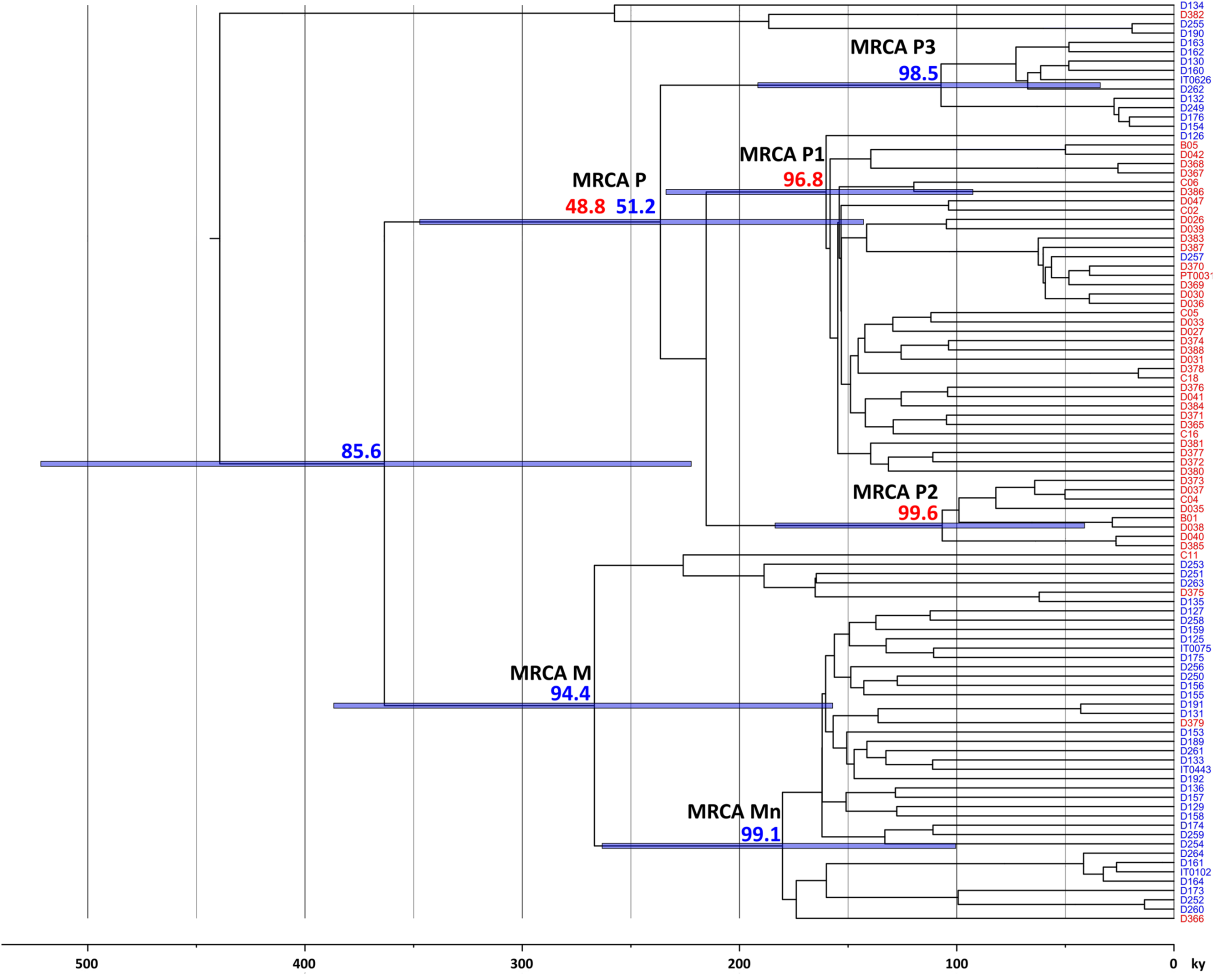
Bayesian phylogeographic analysis restricted to Iberian and Italian samples suggests both eastward and westward ancient olive fly dispersal in the Mediterranean basin. Maximum clade credibility phylogenies for olive fly partial mtDNA. Numbers indicate probabilities of location (in percentage) for key, well-supported (posterior >0.95) nodes (including MRCAs of several clades). Bars represent confidence intervals (95% HPD) for ages. Red—Iberia, Blue—Italy.

To start dissecting the historical processes underlying the observed genetic structure, we used a phylogenetic diffusion model implemented in a Bayesian framework to obtain information regarding the ancestral history of our samples. When applied to the full sample set, this analysis yielded some interesting results (Fig 6). First, common ancestors of the P and M lineages that predominate in the Central and Western Mediterranean basin nowadays were already present in the area hundreds of thousands of years ago. This lends further support to the notion [13] that expansion of the olive fly to the Western Mediterranean is much older than olive tree cultivation. Second, although ancestors of Levantine samples in the 100,000 years BP (yBP) range were apparently from the Levant, the common ancestor of all clade O members—dated ~250,000 yBP—was found to be ~12 times more likely to have been from Italy. Third, there is strong evidence that clades P1 and P2 originated west of the Alps, whereas Mn derives from an Italian ancestor. Since haplotypes belonging to both P1 and Mn are now present from Iberia to Italy, this implies the occurrence of both westwards and eastwards ancient olive fly population movements in the Central and Western Mediterranean basin. Fourth, several clades were found to have mid to high probabilities of having originated in France. However, the calculated dates of all the relevant ancestors are anterior to the last glacial maximum, when paleoclimatic evidence indicates no olive trees would be present in Southern France [24]. This implies that extant populations in the region derive from relatively recent (<12,000–15,000 yBP) migrations from Iberia and/or Italy (where olive trees persisted throughout the glacial periods), suggesting that using only Iberian and Italian samples would provide a more realistic assessment of the ancestors' locations. The results obtained with this analysis (Fig 7) strongly support the notion that clades P1 and P2 originated in Iberia, whereas P3 and M derive from Italian ancestors—and therefore the occurrence of ancient bi-directional migrations between the two peninsulas.

## Discussion

Despite important advances over the past decade, many questions regarding the structure *of B*. *oleae*'s populations in the Mediterranean basin remain open. Several studies point to the existence of at least three different populations, but the geographic limits of their ranges are poorly defined, and the level of differentiation among them is somewhat controversial [7,8,11,12,13,14]. Earlier microsatellite-based studies found either no [10] or limited [11,12] differentiation, whereas whole mitochondrial genome comparisons later pointed to clear separations [13]. Barring different dispersal patterns for males and females, which are unlikely [25], this difference was attributed to the particular mode of inheritance of mtDNA, and the use of complete mtDNA sequences [13]. Indeed, a previous study using only 0.6 kb of mtDNA had failed to detect intra-Mediterranean differentiation [10], in agreement with a previous suggestion that, given the observed level of sequence diversity in *B*.*oleae*, analysis of 2 kb or more of mtDNA would be warranted [23]. We therefore used 3.8 kb (~25% of the mitochondrial genome) in our previous study, which suggested a deep split between Western Iberian and Italic populations [14]. However, the results obtained also suggested that a smaller fraction could still provide good discrimination, prompting our option for the present work (1.8 kb). This option was fully vindicated, as Western Iberian *vs* Italic pairwise F_ST_ values observed were at least eight times those obtained with nuclear microsatellites, demonstrating the superiority of mtDNA in discriminating between these populations.

Application of this strategy to our full sample set and comparison with previously reported sequences resulted in the identification of 77 haplotypes, defined by 88 sequence variants, of which 59 and 56, respectively, are reported for the first time (S1 Table). Fifty four novel haplotypes were identified in the Central and Western Mediterranean samples alone, more than doubling the number of described haplotypes from the region, which by itself represents a significant contribution to the genetic characterization of its *B*. *oleae* populations. The present study also provides an important extension of the region's coverage, particularly at the mtDNA level. Indeed, given previous data suggesting differentiation between Iberian and Italic populations [7,11,12,13,14], the limited information previously available from Southern France (five samples from a single location) represented a major gap [8] that has now been largely filled.

More importantly, the results of our mtDNA haplotype analyses shed a new light onto several aspects of *B*. *oleae*'s population structure in the Mediterranean basin. First, they confirm our previous contention of a deep split between Iberian and Italic populations [14], as clearly indicated by a pairwise F_ST_ value of 0.38 and mitochondrial lineage distributions—dominated (>89%) by P1+P2 in Iberia and M+P3 in Italy. However, they also reveal that this differentiation has a more complex structure than previously recognized. Indeed, both haplotype networks and Bayesian analysis show that lineage P is subdivided, not just in two sublineages, as previously suspected, but in three, with P3 (~19% of Italic sequences) and P2 respectively absent from Iberia and Italy, while P1, which predominates (~73%) in Iberia had only a residual (~4%) presence in Northwestern Italy. The P lineage substructure was therefore found to be geographically correlated and to provide an important contribution to the differentiation between Iberian and Italic populations. This result suggests that the presence of a P (P3) haplotype in Sicily [13] can be regarded as typical of Italy, unlike we had speculated [14], and highlights the importance of using a large enough fraction of mtDNA in phylogeographic studies of *B*. *oleae* [23]. Interestingly, the results also raised the possibility that P lineage substructure contributes to intra-Iberian population differentiation, as a large difference was observed between the frequencies of P2 in Western (25.0%) and Eastern Iberia (8.3%). As for the main questions this study meant to address, the results clearly show that the marked split between Iberian and Italic *B*. *oleae* populations does not result from extant hypothetical geographic limits imposed by the Alps or the Pyrenees, as no differentiation was detected across either of these mountain ranges. More generally, the results suggest there is no clearly defined boundary: different lineage distributions were indeed observed in Southwestern and Southeastern France (as reflected by the SW-SE pairwise F_ST_ value) but, given the evidence for extensive intermixing (*e*.*g*. Fig 3b), this cannot be interpreted as evidence for a sharp boundary. In fact, intermixing was observed to extend at variable levels throughout virtually the whole area under analysis, from Northwestern Italy to Portugal. Together with the apparent patterns of regional lineage distributions (Table 4) and pairwise F_ST_ values (Table 5) along the East-West axis, this raised the possibility of a stepwise variation in population structure. However, a gradual, geographically-correlated, transition was not supported by either RDA (Fig 5) or a Mantel test (not shown). Bayesian phylogeographic analysis of the data, combined with paleoclimatic evidence provides important clues to how this population structure came about. Indeed, the most recent common ancestors (MRCAs) to the P1, P2 and P3 lineages have all been dated to 100,000–200,000 yBP (prior to the Würm glacial period), the first two in Iberia and the latter in Italy. Similarly, MRCAs to the P and M lineages were dated to the Mindel glacial period—the latter likely in Italy and the former having similar probabilities of being from either peninsula. This suggests that the current population structures in Western Europe—including the intermediate one found in Southern France—were likely generated by cycles of glaciation-mediated isolation and bi-directional dispersal and consequent intermixing after the end of the glacial periods.

Importantly, we also found evidence suggestive of intermixing with populations from the Eastern Mediterranean. Indeed, O lineage sequences, typical of the Levantine regions (*e*.*g*. all sequences from the Palestinian samples analysed belong to this lineage) were found in Italy at levels similar to those of lineages typical of Iberia, and even in Eastern Spain. Such intermixing would be consistent with the recent observations of some level of differentiation (but not a clear split) between regions of Turkey around the Levant and close to the Aegean [8] and implies that the source of the olive fly's invasion of California could be located within an area significantly larger than previously thought. Interestingly, Bayesian analysis points to Italy, rather than the Levant, as the more likely source of the O lineage. However, given the lack of comparable information on the genetic make-up of the wide olive-growing area between Italy and the Levant (and also the limited number of O lineage sequences from Italy analysed), this result should be viewed as raising an intriguing hypothesis that needs to be tested in future work. More generally, it is important to emphasize that, given the large uncertainties in dating common ancestors, the notion that glacial cycles played a central role in shaping current olive fly population structure in Western Europe might still be challenged in the future. The overall picture suggested by the present work for the olive fly's population structure in the Mediterranean basin is one of higher levels of lineage homogeneity at the limits of its Mediterranean range (Western Iberia and the Levant—as shown by the Palestinian samples), with variable (possibly distance-related) intermixing throughout most of the range. This picture is similar to that derived from microsatellite analyses [11,12], with at least two important differences: first, it implies the existence of three different dispersion sources, one in the West (in Iberia or, more generally, around the Gibraltar Straits), one in the East (the Levant [26] or, more generally, the fertile crescent [27]) and one in the Italo-Aegean area (or even modern-day Tunisia [7]); second, dispersion was largely human-independent and in both West-to-East and East-to-West directions. Given that Iberia and the Levant have been identified as major long-term olive tree *refugia*, our results also raise the possibility of co-evolution of *B*. *oleae* and its host tree in the Mediterranean.

## Conclusions

Olive fly populations in the Italic peninsula are markedly different from their counterparts in Iberia and the Levant, even though representatives from the mtDNA lineages that predominate in the latter two regions can also be found in Italy. One of the three Mediterranean mtDNA lineages displays geographically-correlated substructure, with the sublineage predominating in Iberia being rare in Italy and vice-versa. The marked differentiation between Iberian and Italic olive fly populations is not due to extant constrains on gene flow imposed by the Alps or the Pyrenees nor, more generally, to any extant sharp boundaries, but likely to the interplay between isolation-mediated differentiation during glacial periods and bi-directional dispersal and consequent widespread population intermixing in the interglacial periods.

## Supporting Information

S1 Table MtDNA haplotypes found in a comparison of olive fly populations from Iberia, France, the Italic peninsula and the Levant. (XLS)
